# AGE DIFFERENCES IN SOCIAL DISCOUNTING AND CHARITABLE GIVING IN A US SAMPLE

**DOI:** 10.1093/geroni/igad104.0097

**Published:** 2023-12-21

**Authors:** Yi Lu, Corinna Löckenhoff

**Affiliations:** Cornell University, Ithaca, New York, United States; Cornell University, Ithaca, New York, United States

## Abstract

Social discounting describes the tendency to show less generosity to more socially distant recipients. With respect to age differences, prior studies (Pornpattananangkul et al., 2019; Lin & Zhang, 2020) found that older adults showed more generosity to distant others (i.e., lower social discounting rates) than younger adults. However, prior research relied on small samples, compared extreme age groups, and focused on Asian countries. To examine generalizability, this pre-registered study examined social discounting and charitable giving in a U.S. adult life-span sample (N = 426, age 18-96, Mage = 50.93, SDage = 19.51, 51% female, 61% non-Hispanic White). The social discounting measure was adapted from Jones and Rachlin (2006) and involved five social distances (1, 5, 10, 50, 100) on a 100-point scale (0 = closest, 100 = most distant). For each social distance, participants were asked how much of $100 they would allocate to themselves versus a person at that distance. They made the same decision for their favorite charity. Contrary to previous findings and our predictions, results revealed a significant positive association between age and social discounting rates (p< .01), such that older adults’ generosity declined more with increasing social distance. Age differences remained robust after controlling for demographics. There were no significant age differences in average giving across social distances and in charitable giving, but, regardless of age, charitable giving was higher among those with lower social discounting rates (p< .001). Findings suggest that age differences in social discounting may be sensitive to cultural context.

